# Development and validation of a disease-specific quality of life measure QLICD-HY (V2.0) for patients with hypertension

**DOI:** 10.1038/s41598-023-39802-2

**Published:** 2023-08-09

**Authors:** Yuxi Liu, Yue Chang, Dandan Wan, Weiqiang Li, Chuanzhi Xu, Chonghua Wan

**Affiliations:** 1 The First Dongguan Affiliated Hospital of Guangdong Medical University, Dongguan, China; 2 School of Humanities and Management, Institute of Health Law and Policy, Guangdong Medical University, Dongguan, China; 3 School of Medicine and Health Management, Center of Medicine Economics and Management Research, Guizhou Medical University, Guiyang, China; 4 Wenzhou Seventh People’s Hospital, Wenzhou, China; 5 The First Affiliated Hospital of Guangdong Pharmaceutical University, Guangzhou, China; 6 School of Public Health, Kunming Medical University, Kunming, China

**Keywords:** Diseases, Health care

## Abstract

The purpose of the present study is to develop and validate the hypertension scale of the Quality of Life Instruments (QoL) for Chronic Diseases system, QLICD-HY (V2.0). The QLICD-HY (V2.0) was developed via a programmed decision method with several focus groups, nominal discussions and pilot testing. The data was collected from 370 hypertensive inpatients and measured their QoL three times before and after treatment. Using correlation, factor analyses, as well as t-tests, the psychometric properties of the scale were assessed with regard to validity, reliability and responsiveness. Correlation and factor analysis supported good construct validity and criterion-related validity when using Short Form 36 as a criterion. Test–retest reliability coefficients for the overall scale score and all domains, with the exception of the psychological and social domain (0.77, 0.78), were greater than 0.80, with a range of 0.77–0.92. The internal consistency for all domains was higher than 0.70. With the exception of the psychological domain and social domain, the overall score and scores for the majority of aspects within each domain underwent statistically significant changes (t-tests) after the treatment. The QLICD-HY (V2.0) has good validity, reliability and responsiveness and can be used as a QoL measure for hypertensive patients.

## Introduction

Hypertension is a common non-communicable disease and the primary risk factor for cardiovascular and cerebrovascular diseases. It not only causes high disability and mortality but also imposes a heavy burden on patients, families and society^1,2^. According to the data released by the World Health Organization, high blood pressure can significantly increase the risk of heart, brain, kidney disease and other diseases, and it is the primary cause of premature death in the world^3^. The Global Burden of Disease Study 2019 estimated that there were approximately 10.85 million deaths due to hypertension worldwide in 2019, accounting for 31% of all deaths^4^. With the ageing of the population and changes in residents’ lifestyles, the number of people with hypertension in China continues to increase and it has become an important public health problem^5^. From 2012 to 2015, the crude prevalence rate of hypertension amongst residents aged 18 and over in China was 27.9% (the standardized rate was 23.2%), and the prevalence was on the rise compared with the past^4^. The deaths attributable to hypertension amongst Chinese residents increased from 1.22 million in 1990 to 2.599 million in 2019, the increase rate was 112.72%; the mortality attributable to hypertension increased from 103.25/10^5^ in 1990 to 182.79/10^5^ in 2019, the increase rate was 77.04%^6^.

With the improvement of people’s health needs, the medical model has changed to a biological-social-psychological model. Quality of life (QoL) has been gained more attention in medical field, and has increasingly become a focus for research and application in many fields. When evaluating the therapeutic effect of a disease, not only should biological indicators be used to evaluate the physical function, but also psychological and social indicators to evaluate overall function (i.e. QoL). QoL is a complex concept that is interpreted and defined differently within and between disciplines. In this paper, QoL is individuals’ perception of their position in life in the context of the culture and values systems in which they live and in relation to their goals, expectations, standards and concerns^7^. Compared with traditional indicators (such as cure rate and mortality), QoL is more suitable for the health evaluation of patients with chronic diseases for it reflect the new medical model and also “patient-centered” orientation. Given the fact that most patients with hypertension require long-term treatment, it becomes increasingly important to investigate the effect of treatment on hypertension patients’ QoL^8^. There are numerous studies on the QoL of patients with hypertension with a lot of them being on influencing factors such as age, sex, alcohol consumption^9–11^, only a few studies being on the instrument of QoL for hypertension. Although generic instruments for measuring QoL are generally used in both the general population and patients with hypertension, such as Brief Version of World Health Organization Quality of Life^12^, short- Form-36^13^, and European Quality of Life-5 Dimensions^14^, they do not capture the symptoms and side effects specific to hypertension. In contrast, disease-specific instruments such as the short form of hypertension quality of life Questionnaire (MINICHAL)^15^, Cambridge pulmonary hypertension outcome review (CAMPHOR)^16^ and Development of the pulmonary arterial hypertension symptoms and impact (PAH-SYMPACT)^17^ are focused on symptoms and signs that reflect the status of hypertension and more efficient than generic questionnaires. Thus, a more specific QoL measure, developed particularly to assess hypertension problems, would be useful in assessing QoL and evaluating whether treatment is successful or not.

To the best of the author’s knowledge, no scale for hypertension was developed based on the modular approach (a general module plus specific modules). In addition, no Chinese version of any of these instruments was available for use in patients with hypertension in China^8^. To fill this gap, the author developed a system of QoL Instruments for Chronic Disease (QLICD), which included a general module (QLICD-GM) and a disease-specific module for each disease considered. The QLICD (V1.0) contained one general module and nine disease-specific modules including hypertension (QLICD-HY), coronary heart disease (QLICD-CHD), chronic obstructive pulmonary diseases(QLICD-COPD), chronic pulmonary heart diseases(QLICD-CPHD), bronchia asthma (QLICD-BA), chronic gastritis (QLICD-CG), peptic ulcer (QLICD-PU), irritable bowel syndrome(QLICD-IBS), diabetes mellitus(QLICD-DM), which are widely used in some studies in China^18^. However, some problems were also found or reported in the clinical application of the QLICD (V1.0). For example, the structure of the scale was not completely reasonable, there was imprecision in reliability estimation and questions surrounding the validity of the test results. To solve these problems, the author developed the latest version of the system QLICD (V2.0), which contained 32 chronic disease-specific scales^19^. The present study developed the specific module for patients with hypertension, and then combined it with the general module that had been developed to form the hypertension instrument QLICD-HY (V2.0). In this paper, the author aimed to report on the development and validation of the QLICD-HY (V2.0) instrument.

## Materials and methods

### Development of the QLICD-HY (V2.0)

Development of the QLICD-HY (V2.0) was based on the construction of the general module (QLICD-GM) and the specific module development across several domains for hypertension patients.

#### Construction of the general module (QLICD-GM)

The method of the programmed decision was used for item selection, and a multi-step process was formed. Firstly, the author had to establish a research team (nominal and focus groups) to define OoL measurement concepts and propose an item pool. Secondly, the author had to screen items to form a primary scale. Thirdly, the author had to screen pre-survey items to form a test version scale. Finally, the author had to evaluate the scale and form the formal scale.

The nominal group consisted of 16 individuals, including seven researchers (two QoL researchers, two sociologists, two psychologists and one epidemiologist), six doctors, two nurses, and one medical manager for chronic diseases, which proposed the item pool by reviewing the literature and referring to existed QoL scales. The focus group was composed of 10 experts, including five researchers (two in QoL, one in epidemiology, one in sociology and one in psychology), which proposed the conceptual framework of QoL and refined items proposed by the nominal group. Both qualitative methods such as in-depth interviews, focus group discussions and quantitative methods such as variation, correlation and factor analyses were used in the item selection process. In the end, 28 items were selected to form the QLICD-GM, including three domains (nine items in the physical domain, 11 items in the psychological domain and eight items in the social domain), and each item was scored on a five-point Likert scale.

**Figure 1:**
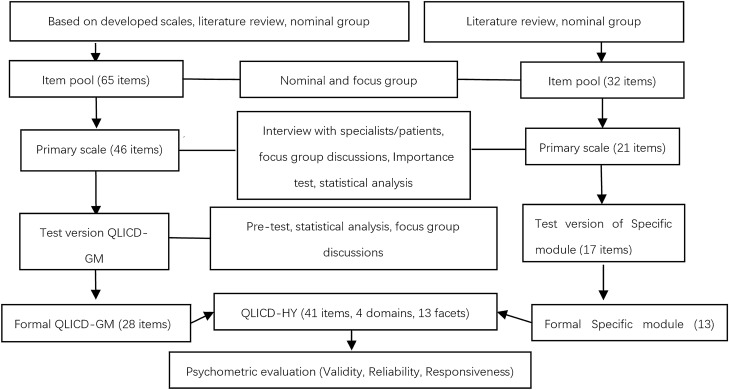
Steps towards development and validation procedure of QLICD-HY (V2.0).

The scale was revealed to have a good validity^20–22^ based on data from 1672 individuals with 11 chronic diseases: 185 in hypertension, 163 in chronic gastritis, 170 in peptic ulcer, 124 in chronic obstructive pulmonary disease, 242 in diabetes, 140 in osteoarthritis, 100 in rheumatoid arthritis, 143 in systemic lupus erythematosus, 100 in stroke, 141 in prostatic hyperplasia and 164 in chronic renal failure. The primary steps were summarised in Fig. 1.

#### Construction of the hypertension-specific module

Using the similar multi-step procedure as the general module, 21 items were selected from the 32-item pool of the hypertension-specific module based on literature review, nominal group/focus group discussion and in-depth interviews.

After pre-test and two-stage screenings, the final specific module of 13 items (coded HY1-HY13) was formed including four facets of cerebrovascular symptoms (CES), cardiovascular symptoms (CAS), treatment side effects (TSE) and special effects on mentality and life (EML) (Fig. 1). In brief, the following three statistical procedures were used to screen the items based on pre-test data at this stage.

A. Variation procedure. The standard deviation (SD) of the scores for each item was calculated and the items with smaller SD (< 1.10) were deleted.
B. Correlation procedure. The Pearson’s correlation coefficients between the score of each item and the sum score of its own domain were computed and the items with smaller correlation coefficients (< 0.50) were deleted.
C. Patients item importance rating procedure. The patients were asked to score the importance of each item using a 0–100 score system (0 = extremely unimportant and 100 = extremely important). The items with score < 65 were deleted.

### Validation of the QLICD-HY (V2.0)

#### Data collection

The QLICD-HY (V2.0) was developed by combining the newly created hypertension-specific module with the general module QLICD-GM. It was used to investigate and assess hypertension patients in the field. The survey was conducted at the First Affiliated Hospital of Kunming Medical University in Kunming, Dalang hospital and Shilong Boai hospital in Dongguan, China. We enrolled participants with hypertension at any stage who were: (1) be able to read and write words with assistance and understand the content of the questionnaire; (2) volunteer to participate in the assessment and be able to provide written informed consent. The exclusion criteria comprised: (1) illiterate; (2) unconscious and unable to clearly express their feelings; (3) serious diseases. The study’s objective and significance were described to the participants by the investigators (doctors and medical post-graduate students), who received their informed consent. The survey institution’s ethics committee gave its approval to the research procedure and informed consent form.

At the time of admission into the hospital, all 370 respondents completed the questionnaires. A randomly selected subsample of 121 patients also took part in a follow-up evaluation after one or two days after being hospitalised to determine test–retest reliability, considering their QoL not change in nature because treatments has not been effected in these days.

In addition, after approximately a week of therapy, 321 patients who were accessible at the third scheduled evaluation time point completed the measures at discharge to gauge responsiveness. The investigators promptly verified each response to make sure it was complete. If missing values were discovered, the questionnaire was given back to the patients so they could fill out the blanks.

#### The scoring method of the QLICD-HY (V2.0)

On the basis of the collected data, the raw scores of items, domains, and the overall scale were determined. Each item on the QLICD-HY is scored on a five-point Likert scale: not at all, slightly, moderately, very much and extremely. The positively expressed items were assigned a score between 1 and 5, whilst the negatively stated items were reverse-coded. Each domain score was derived by aggregating the item scores inside the domain. The total score on the scale was the sum of the domain scores. All domain scores were linearly translated to a 0–100 scale for the purposes of comparison using the following formula: SS = (RS-Min) 100/R, where SS represents the standardised score, RS represents the raw score, Min represents the minimum score and R represents the range of scores.

#### Reliability

In the present study, internal consistent reliability and test–retest reliability were used to evaluate the scale’s reliability. Internal consistency was determined using Cronbach’s alpha coefficient for each domain, and test–retest reliability was determined using Pearson’s correlation coefficient r and intra-class correlation (ICC). A Cronbach’s alpha coefficient between 0.70 and 0.95 is viewed as sufficient evidence of internal consistency in scale development^23^. Sehunemann suggested that test–retest reliability between 0.73 and 0.95 was sufficient^24^.

#### Validity

Due to the lack of a consensus gold standard for evaluating the QOL of hypertension, the author decided to utilise the Chinese version of the SF-36 as the criteria for evaluating criterion-related validity. The criterion-related validity was assessed by correlating the corresponding domains of QLICD-HY and SF-36, and also the convergence validity and discriminant validity was tested by this multi-trait scaling analysis^23^ simultaneously. There are two validity criteria: (1) when the item-domain correlation is 0.40 or higher, it supports convergence validity; (2) discriminant validity is revealed when item-domain correlation is higher than that with other domains.

Construct validity was evaluated by calculating the Pearson correlation coefficient (r) among items and domains considering that these scores have been transformed to 0–100 quantitative data with normal distribution, as well as by confirmatory factor analysis using structural equation modeling. The author calculated Pearson’s correlation coefficient *r* between items and domains with a minimum threshold of 0.40^23,25^. Concerning structural equation modelling, CFI (comparative fit index)TLI (Tucker–Lewis index), RMSEA (root-mean-square error of approximation) and SRMR (standardized root mean square residual) are indices recommended as sensitive to model misspecification^26^, with the CFI and TLI with values greater than 0.90 and RMSEA, SRMR less than 0.08 reflecting a good fit of the model to the data.

#### Responsiveness

The responsiveness was evaluated by comparing the mean difference between the first and third evaluations (before and after treatments). The paired t-test was employed to evaluate responsiveness with the calculation of the standard responsiveness mean (SRM). The SRM was the ratio of the difference between before and after treatments to the standard deviation of the difference. According to Husted^27^, an SRM above 0.8 indicated good responsiveness, an SRM between 0.5 and 0.2 revealed moderate responsiveness, and an SRM below 0.2 indicated a poor response.

### Ethics statement

The study protocol and the informed consent form were approved by the Institutional Review Board of Guangdong Medical University (PJ2013037) and performed in accordance with the World Medical Association Declaration of Helsinki for ethical principles for medical research involving human subjects. All procedures performed in studies involving human participants were in accordance with the ethical standards of the institution or practice at which the studies were conducted. The participants provided their written informed consent to participate in this research.

## Results

### Socio-demographic characteristics of the sample

The age range for 370 patients with hypertension was 29 to 88 years old, with a median age of 67 years and a mean age of 64.8 + 10.6 years. 160 (43.2%) were male, and 134 (63.3%) had finished primary school. The majority of participants (88.4%) were married, and 43 cases (11.6%) were widowed. Public insurance was the most common type of medical insurance amongst the study population (78.4%). Workers who performed manual labour in factories made up 34.1% of the workforce, followed by farmers with 21.1% (see Table 1).

**Table 1:** Socio-demographic characteristics of the sample (n = 370).

Characteristics	N	%	Characteristics	N	%
Gender			Marital status		
Male	160	43.2	Married	327	88.4
Female	210	56.8	Others	43	11.6
Age			Medical insurance		
< 30	1	0.3	Self-paid	20	5.4
30–39	20	5.4	Partly public insurance	50	13.5
40–49	58	15.7	Public insurance	290	78.4
50–59	105	34			
≥ 60	186	44.6	Occupation		
Education			Factory worker	126	34.1
Primary school	134	63.3	Farmer	78	21.1
High school	72	19.5	Teacher	12	3.2
College or higher	59	15.9	Officer/manager	61	16.5
			Others	87	23.5

### Reliability

Three approaches were used to examine the reliability of the scale: test–retest, ICC and internal consistency (see Table 2 for details). The test–retest correlation coefficients (r) for the four domains and 13 dimensions of QLICD-HY ranged from 0.49 to 0.92, with r = 0.90 for the overall scale and r = 0.77 for SOD as the lowest amongst the four exclusive domains. The differences in domain and facet scores between the first and second assessments were not statistically significant (*P* > 0.05). ICC results based on the criterion of the absolute agreement for a single measure were remarkably close to Pearson’s correlation coefficients *r*. Cronbach’s α coefficient for these four domains was between 0.70 and 0.88, which revealed good internal consistency reliability (Table 2).

**Table 2:** Reliability, floor and ceiling effects of the quality of life instrument QLICD-HY (V2.0).

Domains/facets	Internal consistency coefficient α	Test–retest reliability correlation *r*	ICC (95%CI)
Physical domain (PHD)	0.70	0.85	0.92 (0.88–0.96)
Basic physiologic functions (BPF)	0.45	0.80	0.89 (0.85–0.93)
Independence (IND)	0.87	0.69	0.81 (0.77–0.85)
Energy and discomfort (EAD)	0.51	0.56	0.72 (0.68–0.76)
Psychological domain (PSD)	0.84	0.78	0.87 (0.83–0.91)
Cognition (COG)	0.41	0.80	0.88 (0.84–0.92)
Emotion (EMO)	0.84	0.62	0.76 (0.72–0.80)
Will and personality (WIP)	0.53	0.79	0.88 (0.84–0.92)
Social domain (SOD)	0.76	0.77	0.87 (0.83–0.91)
Interpersonal communication (INC)	0.65	0.65	0.78 (0.73–0.82)
Social support and security (SSS)	0.62	0.62	0.75 (0.71–0.79)
Social role (SOR)	0.29	0.49	0.66 (0.62–0.70)
Sub-total (QLICD-GM)	0.87	0.83	0.90 (0.86–0.94)
Specific domain (SPD)	0.73	0.92	0.95 (0.91–0.99)
Cerebrovascular symptoms(CES)	0.68	0.88	0.93 (0.89–0.97)
Cardiovascular symptoms (CAS)	0.61	0.75	0.85 (0.81–0.89)
Treatments side effects (TSE)	0.41	0.80	0.88 (0.84–0.92)
Special Effects on Mentality and Life (EML)	0.17	0.60	0.75 (0.71–0.79)
Total (TOT)	0.88	0.90	0.94 (0.90–0.98)

*ICC* Intra-class correlation, *CI* Confidence interval.

### Construct validity

Strong correlations between items and their own domains/facets subscales were found using correlation analyses (the most of correlation coefficients were higher than 0.5), while weak associations between items and other domains/facets were also found (see Table 3 in detail). For example, correlation coefficients between items of GPH1-GPH9 and physical function domain (in bold) are greater than those across domains. Similarly, correlation coefficients between items of GSO1–GSO8 and social function domain (in bold) are greater than those across domains. These associations were consistent with the conceptual theoretical constructs.

**Table 3:** Correlation coefficients r among items and domains of QLICD-HY (V2.0) (n = 370).

Code	Items brief description	Physical	Psychological	Social	Specific
GPH1	Appetite	0.51**	0.35**	0.25**	0.20**
GPH2	Sleep	0.45**	0.15**	0.10**	0.27**
GPH3	Sexual function	0.47**	0.17**	0.09**	0.17**
GPH4	Excrement	0.46**	0.21**	0.25**	0.24**
GPH5	Pain	0.55**	0.18**	0.37**	0.32**
GPH6	Daily activities	0.59**	0.43**	0.43**	0.16**
GPH7	Work	0.66**	0.38**	0.44**	0.16**
GPH8	Walk	0.58**	0.36**	0.40**	0.13**
GPH9	Fatigue	0.55**	0.33**	0.17**	0.33**
GPS1	Attention	0.56**	0.55**	0.47**	0.36**
GPS2	Memory deterioration	0.41**	0.24**	0.02**	0.33**
GPS3	Joy of life	0.14**	0.37**	0.31**	0.09**
GPS4	Restless	0.31**	0.61**	0.28**	0.32**
GPS5	Family burden	0.18**	0.63**	0.46**	0.12**
GPS6	State of health	0.26**	0.72**	0.43**	0.18**
GPS7	Depression	0.36**	0.81**	0.51**	0.23**
GPS8	Disappointment	0.40**	0.81**	0.54**	0.27**
GPS9	Fear	0.30**	0.76**	0.45**	0.20**
GPS10	Positive attitude	0.35**	0.63**	0.63**	0.18**
GPS11	Termagancy	0.28**	0.73**	0.40**	0.29**
GSO1	Social contact	0.43**	0.52**	0.76**	0.19**
GSO2	Family relationship	0.10**	0.23*	0.50**	0.18*
GSO3	Friend relationship	0.12**	0.14	0.49**	0.12*
GSO4	Family support	0.23**	0.42*	0.71**	0.10**
GSO5	Other people’s care	0.28**	0.39**	0.72**	0.13**
GSO6	Economic hardship	0.17**	0.55**	0.63**	0.15**
GSO7	Labor status	0.32**	0.40**	0.55**	0.22**
GSO8	Family role	0.40**	0.46**	0.71**	0.12**
HY1	Headache	0.20**	0.16**	0.02**	0.53**
HY2	Dizzy	0.30**	0.16**	0.06**	0.58**
HY3	Tinnitus	0.26**	0.22**	0.14**	0.57**
HY4	Heart palpitations	0.27**	0.22**	0.12**	0.62**
HY5	Shortness of breath	0.30**	0.18**	0.14**	0.59**
HY6	Swelling in ankle/legs	0.06**	0.05**	0.05**	0.37**
HY7	Numb/immobile limb	0.25**	0.30**	0.17**	0.57**
HY8	Chest/shoulder/back pain	0.22**	0.17**	0.13**	0.57**
HY9	Dry mouth/ irritable cough	0.22**	0.27**	0.15**	0.52**
HY10	Blurred vision	0.22**	0.01**	0.08**	0.48**
HY11	Bothered by too much drug	0.09**	0.18**	0.15**	0.39**
HY12	Facial redness	0.16**	0.42**	0.40**	0.40**
HY13	Adapt to life style change	0.03**	0.11**	0.19**	0.07**

**Figure 2:**
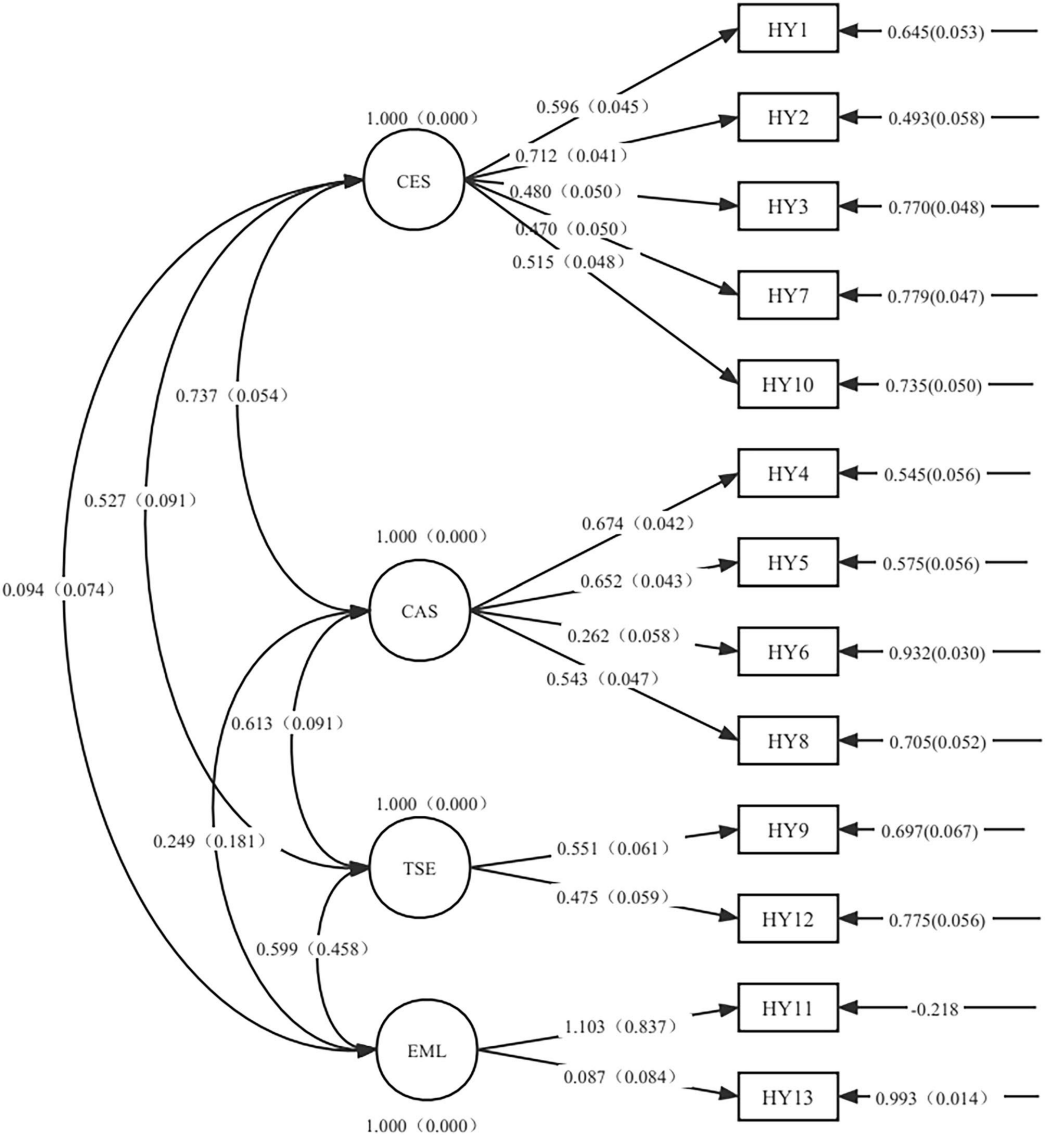
The structure of the specific module of QLICD-HY by structural equation modeling.

Structural equation modeling showed that the structure of the specific module of the QLICD-HY was consistent with the conceptual theoretical construct (four facets), with goodness of fit Chi-square $\chi^{2}$ = 192.007 (*P* < 0.001), Tucker–Lewis index (TFI) = 0.941, comparative fit index (CFI) = 0.938, root mean square error of approximation (RMSEA) = 0.078, standardized root mean square residual (SRMR) = 0.066. See Table 4 and Fig. 2 in detail.

**Table 4:** Structure of the specific module of the QLICD-HY confirmed by SEM (*n* = 370).

Facets	Items	Path coefficients	SE	Z	P	Standardized path coefficients
CES (cerebrovascular system symptoms)	HY1	1.000	0.000			0.596
	HY2	1.183	0.121	9.766	< 0.001	0.712
	HY3	0.775	0.116	6.684	< 0.001	0.480
	HY7	0.688	0.104	6.631	< 0.001	0.470
	HY10	0.869	0.123	7.065	< 0.001	0.515
CAS (cardiovascular system symptoms)	HY4	1.000	0.000			0.674
	HY5	0.934	0.101	9.239	< 0.001	0.652
	HY6	0.305	0.075	4.085	< 0.001	0.262
	HY8	0.761	0.097	7.811	< 0.001	0.543
TSE (treatment side effects)	HY9	1.000	0.000			0.551
	HY12	0.693	0.112	6.186	< 0.001	0.475
EML (special effects on mentality and life)	HY11	1.000	0.000			1.103
	HY13	0.101	0.167	0.606	0.544	0.087

### Criterion-related validity

The Chinese version of the SF-36 was employed to provide data for evaluating the QLICD-HY criterion-related validity. The correlation coefficients between the QLICD-HY and the SF-36 domain scores were expressed in Table 5. It can be seen from Table 5 that, overall, the correlations between the same and similar domains of the QLICD-HY and the SF-36 are higher than those between different and non-similar domains. For instance, the coefficients between physical domain of the QLICD- HY and the SF-36 was 0.50, and that between physical domain of the QLICD- HY and vitality of the SF-36 was 0.53, which were higher than all the other coefficients in this row. Obviously, the physical domain of the QLICD- HY was the same or similar to physical function and vitality of the SF-36. Additionally, the psychological domain of the QLICD-HY and mental health domain of the SF-36 had the largest correlation coefficient (0.67) in this row. These correlations confirm the criterion-related validity and also demonstrate the convergent and divergent validity to a reasonable degree.

**Table 5:** Correlation coefficients among domains scores of QLICD-HY (V2.0) and SF-36 (n = 370).

QLICD-HY	SF-36							
	Physical function	Role-physical	Body pain	General health	Vitality	Social function	Role-emotional	Mental health
Physical domain	**0.50**	0.36	0.47	0.43	**0.53**	0.30	0.38	0.36
Psychological domain	0.39	0.34	0.36	0.19	0.47	0.44	0.39	**0.67**
Social domain	0.32	0.38	0.23	0.03	0.29	**0.42**	0.35	0.46
The specific module	0.27	0.23	**0.51**	0.35	0.40	0.22	0.27	0.30

There was a significant correlation at the level of 0.05 for all correlation coefficients.

Correlations in bold were that for similar domains.

### Responsiveness

According to Table 6, all domains of the scale were statistically significant (*P* < 0.05). SRM in various domains ranged from 0.22 to 0.85, with 0.77 and 0.85 for the specific module and overall scale, respectively. Except for psychological and social domains, other domains of SRM were all above 0.70. It could be noted that the QLICD-HY had good responsiveness.

**Table 6:** Responsiveness of the quality of life instrument QLICD-HY (V2.0) (n = 370).

QLICD-HY	Before treatment		After treatment		Differences		*t*	*p*	SRM
	Mean	SD	Mean	SD	Mean	SD			
Physical domain	67.99	13.28	76.39	13.00	8.75	10.52	14.90	0.001	0.83
Basic physiologic functions	60.30	14.23	67.38	12.98	7.16	12.36	10.38	0.001	0.58
Independence	88.15	20.86	88.70	21.42	0.59	13.02	0.82	0.412	0.05
Energy and discomfort	53.14	24.06	75.93	20.08	24.18	25.26	17.14	0.001	0.96
Psychological domain	73.31	16.72	78.16	14.91	3.78	10.55	6.42	0.001	0.36
Cognition	64.39	22.61	65.30	21.96	1.79	14.13	2.27	0.024	0.13
Emotion	74.03	19.08	80.48	16.60	4.92	12.16	7.25	0.001	0.40
Will and personality	79.72	20.81	82.90	19.42	1.75	14.36	2.18	0.030	0.12
Social domain	81.34	14.37	84.57	13.07	2.03	9.41	3.87	0.001	0.22
Interpersonal communication	82.34	14.36	86.00	13.40	2.54	12.65	3.60	0.001	0.20
Social support and security	84.59	17.58	87.66	16.32	1.27	11.46	1.98	0.048	0.11
Social role	74.96	21.43	77.80	20.39	2.41	14.79	2.92	0.004	0.16
Sub-total (QLICD-GM)	73.02	12.00	78.46	11.54	4.92	7.55	11.68	0.001	0.65
Specific domain	74.00	13.72	84.57	11.68	10.63	13.81	13.78	0.001	0.77
Cerebrovascular system symptoms	68.04	19.87	80.26	13.76	13.30	17.82	13.37	0.001	0.75
Cardiovascular system symptoms	76.38	19.22	90.30	13.96	13.90	19.19	12.97	0.001	0.72
Treatment side effects	86.45	18.24	91.19	15.32	4.24	18.22	4.17	0.001	0.23
Special effects on mentality and life	71.72	21.22	77.29	17.87	3.82	17.15	3.98	0.001	0.22
Total (TOT)	73.42	10.74	80.20	10.54	6.43	7.59	15.17	0.001	0.85

## Discussion

This research focuses on the development and validation of the QLICD-HY (V2.0), a new specific QoL instrument for patients with hypotension. The author developed the second version of QLICD-HY by combing the improved general module QLICD-GM and the developed specific module for hypertension. The general module QLICD-GM capture general QoL in patients with various chronic disease such as chronic obstructive pulmonary disease (COPD) and peptic ulcer, whilst disease-specific modules capture the aspects of QoL that distinguish the different diseases. The content was developed from focus group discussion, in-depth interviews with hypertension patients, pre-testing and psychometric evaluation. The final QLICD-HY (V2.0) was comprised of 41 items, including 28 items of QLICD-GM and 13 items of specific modules, making it concise and usable in research and clinical settings. Therefore, QLICD-HY is different from other QoL instruments for hypertension.

On the basis of the first version of QLICD-GM, the second version of QLICD-GM has undergone various enhancements to strengthen its readability and accessibility^21^. The evaluation of the QLICD-HY (V2.0) indicates that it is a reliable and valid disease-specific instrument. In this research, the internal consistency reliability and the test–retest reliability were used to determine the QLICD-HY reliability^18^, with the results indicating that it has a high degree of reliability. The findings of the test–retest reliability analysis reveal the stability of this instrument, which forms the basis for the evaluation of responsiveness to change.

To demonstrate validity, correlation analysis and confirmatory factor analysis by SEM were used, with the results confirming overall good validity. Correlation analysis revealed weak correlations between items and other domains/facets, whereas there were strong associations between items and their own domains/facets. The validity of the construct was also further confirmed by structural equation modeling, which revealed good fit from the data corresponded with the theoretical constructs of the instrument^21^. Comparable SF-36 domains suggest that the QLICD-HY (V2.0) has good criterion-related validity.

In general, responsiveness evaluation methods can be split into two categories: internal and external^27,28^. The present study focuses on internal responsiveness with the notion that a sensitive instrument should detect changes in response to therapies when testing post-treatment. In this research, the paired t-test was utilised to assess the mean response before and after therapy, along with indicators of responsiveness such as SRM. SRM is a good indicator for estimating effect size, with values of 0.20, 0.50, and 0.80 corresponding to small, moderate and large responsiveness, respectively^27,28^. As revealed in Table 6, all domains changed significantly after therapy, which appears to have good responsiveness.

The quality of life has strong cultural dependence; thus, it is necessary to develop a measuring scale with Chinese cultural characteristics. In China, despite the fact that several scales were constructed in accordance with Chinese culture and exhibited acceptable psychometric qualities^29–31^, there were still deficiencies. Initially, these scales were not established systematically using the modular approach (combination of the general module and specific modules), and therefore cannot be used to compare different diseases. Secondly, these scales lacked a unified standard. In addition, they did not adequately consider the influence of pharmacological side effects on patients, and the majority of them were solely utilised in traditional Chinese medicine. Therefore, a scientific, reasonable, reliable and appropriate QOL assessment scale was required for Chinese hypertension patients. In the present study, the QLICD-HY was created by combining the general module of a recognised and well-established system of QOL instruments for chronic diseases (QLICD) with a newly built hypotension-specific module. In comparison to other scales in China, the QLICD-HY not only demonstrates superior validity, reliability and responsiveness, but also the capacity to evaluate QOL across diseases using the general module, displaying both generic and specific qualities. From the structure of the scale, it is comprised of a moderate number of items with a clear hierarchical structure (items → facets → domains → overall) so that analysis of scores may be conducted not only at the domain and overall levels but also at the different facet levels (13 in total) to discover subtle changes. In addition, QLICD-HY has the characteristics of Chinese culture and adds entries to traditional Chinese culture in terms of appetite, sleep and family affection. Thus, QLICD-HY (V2.0) with Chinese cultural characteristics by means of the modular method is both comparable and targeted.

It is worth acknowledging that the present study was also subject to a few constraints. Firstly, the sample size in the present study is not sufficient for the general module’s factor analysis by SEM, and thus it was not carried out here. Secondly, the participants in the present study were chosen from hospitals’ inpatient populations. To determine whether the instrument is generalisable to other contexts and demographics, such as outpatients at a nearby clinic or community, further research is required.

## Conclusion

In conclusion, combining the well-recognised general module of chronic diseases with the specific module of hypertension, the second edition of QLICD-HY was developed within the context of Chinese culture. It has good validity, reliability and responsiveness, and may be used to assess the QOL of Chinese hypertension patients. This instrument and its future improvements may have the potential to be applied in other countries to improve the measurement of QOL for hypertension patients if the findings are replicated in more general study groups.

### Supplementary Information

Supplementary Information 1.

Supplementary Information 2.

## Data Availability

The raw data supporting the conclusions of this article will be made available by the authors, without undue reservation.
