# Supplementary material for: Development and validation of a disease-specific quality of life measure QLICD-HY (V2.0) for patients with hypertension
Source: Sci Rep. 2023 Aug 9;13:12935. doi: 10.1038/s41598-023-39802-2 (PMC10412614; doi:10.1038/s41598-023-39802-2)
Supplement: Supplementary file 1 — Supplementary Information 1. [file 41598_2023_39802_MOESM1_ESM.pdf]

Quality of Life Instruments for Chronic Diseases – Hypertension Scale QLICD-HY（V2.0）

Hospital Number____________Number ________

First Name________________ Age________

Gender: (1) Male (2) Female

Ethnicity: (1) Han (2) Yi (3) Bai (4) Hui (5) Other

Occupation: (1) worker (2) farmer (3) teacher (4) cadre (5) individual (6) other

Marital status: (1) unmarried (2) married (3) divorced (4) widowed

Education level: (1) primary school (2) junior high school (3) high school or technical secondary school (4) college (5) bachelor degree or above

Family economic situation: (1) poor (2) medium (3) good

Annual net household income per capita:________Yuan

Forms of medical treatment: (1) self-payment (2) social medical insurance (urban employee medical insurance) (3) commercial medical insurance (4) cooperative medical care

Number of times to fill in the form:

Date of Completion: Year Month Day

(The following items are filled in by the doctor)

Treatment hospitals_________________ Treatment department_________________

Clinical diagnosis___________________Clinical staging_________________

Treatment________________________ Clinical classification_________________

Treatment effect: (1) Healing (2) Effective (3) Improvement (4) No change (5) Deterioration (6) Other

Vetting Doctor (Signed)_______________ Review date________________

Guangdong Medical College   Kunming Medical University

Quality of Life Instruments for Chronic Diseases – Hypertension Scale QLICD-HY（V2.0）

INSTRUCTION: This questionnaire helps doctors know your feeling about your health condition in the past week. Your answers will help them choose the appropriate treatment and rehabilitation strategy. There is no right or wrong answer. Please read the following questions carefully, and circle the number most relevant how your feeling. You may choose the answer closest to your true feeling in case you are not sure how to answer the question. The information that you provide will remain strictly confidential.

For example: Do you find life fun?

If you find life more enjoyable, make a circle at "4" as follows.

Not at all	A little	Moderately	Very much	Extremely
1	2	3	④	5

Physical Function

	The past week	Not at all	A little	Moderately	Very much	Extremely
GPH1	Do you have a good appetite?	1	2	3	4	5
GPH2	Do you sleep well?	1	2	3	4	5
GPH4	Is your stool normal?	1	2	3	4	5
GS02	Do you have a good relationship with your family?	1	2	3	4	5
GS03	How good are you with your friends?	1	2	3	4	5

© 版权所有

Physical Function

	The past week	Not at all	A little	Moderately	Very much	Extremely
GPH3	Do you feel sick or treated to affect your sexual function?	1	2	3	4	5
GPH5	Do you have pain or other uncomfortable feelings?	1	2	3	4	5
GPH9	Do you feel tired easily?	1	2	3	4	5
GPS2	Is the disease causing your memory to deteriorate?	1	2	3	4	5
GPS3	Do you find life fun?	1	2	3	4	5

Psychological Function

	The past week	Not at all	A little	Moderately	Very much	Extremely
GPS4	Have you felt nervous and anxious?	1	2	3	4	5
GPS5	Have you thought of yourself as a burden to your family?	1	2	3	4	5
GPS6	Are you worried about your health getting worse?	1	2	3	4	5
GPS7	Are you feeling down or sad?	1	2	3	4	5
GPS8	Are you pessimistic?	1	2	3	4	5
GPS9	Are you afraid of your illness?	1	2	3	4	5
GPS11	Has the illness soured your temper (character)?	1	2	3	4	5
GS06	Did the illness and treatment cause financial hardship in your family?	1	2	3	4	5
GS07	Have illness and treatment affected your status or role in work or labor?	1	2	3	4	5

© 版权所有

Social Function

	The past week	Not at all	A little	Moderately	Very much	Extremely
GPH6	Can you manage your daily life (e.g., eating, dressing, washing, going to the toilet)?	1	2	3	4	5
GPH7	Can you work (e.g. housework, work, farming, etc.)?	1	2	3	4	5
GPH8	Can you walk independently?	1	2	3	4	5
GPS1	Can you concentrate when doing things?	1	2	3	4	5
GPS10	Are you able to take a positive and optimistic view of your illness?	1	2	3	4	5
GS01	Can you relate to others as you did before you got sick?	1	2	3	4	5
GS04	Can you get care or support from your family?	1	2	3	4	5
GS05	Can you have care or support from someone outside your family?	1	2	3	4	5
GS08	Can you assume the appropriate family roles (e.g. parents, children, couples)?	1	2	3	4	5

Specific Module

The past week		Not at all	A little	Mode rately	Very much	Extre mely
HY1	Do you have a headache?	1	2	3	4	5
HY2	Do you have dizziness?	1	2	3	4	5
HY3	Do you have tinnitus?	1	2	3	4	5
HY4	Are you flustered?	1	2	3	4	5
HY5	Do you have shortness of breath or shortness of breath (difficulty breathing)?	1	2	3	4	5

© 版权所有

HY6	Do you have edema of the lower extremities or ankles?	1	2	3	4	5
HY7	Do you have weakness, numbness or mobility on one side?	1	2	3	4	5
HY8	Do you have chest pain or shoulder and back pain?	1	2	3	4	5
HY9	Do you have dry mouth or irritating dry cough after taking the medicine?	1	2	3	4	5
HY10	Do you have vision loss or blurred vision?	1	2	3	4	5
HY11	Are you bothered by the need to take medication or take your blood pressure regularly?	1	2	3	4	5
HY12	Do you experience facial redness and heat after taking the medicine?	1	2	3	4	5
HY13	Can you adapt to dietary restrictions due to illness or lifestyle changes such as quitting smoking?	1	2	3	4	5

© 版权所有
