# Supplementary material for: Development and validation of a disease-specific quality of life measure QLICD-HY (V2.0) for patients with hypertension
Source: Sci Rep. 2023 Aug 9;13:12935. doi: 10.1038/s41598-023-39802-2 (PMC10412614; doi:10.1038/s41598-023-39802-2)
Supplement: Supplementary file 2 — Supplementary Information 2. [file 41598_2023_39802_MOESM2_ESM.pdf]

高血压患者生命质量测定量表

QLICD-HY（V2.0）

住院号______________ 编号_____________

姓 名______________ 年龄_____________

性别：①男 ②女

民族： ①汉族 ②彝族 ③白族 ④回族 ⑤其它

职业： ①工人 ②农民 ③教师 ④干部 ⑤个体 ⑥其它

婚姻状况：①未婚 ②在婚 ③离婚 ④丧偶

文化程度：①小学 ②初中 ③高中或中专 ④大专 ⑤本科及以上

家庭经济状况： ①差 ②中 ③好

家庭年人均纯收入：____ 元

医疗形式：①自费 ②社会医疗保险（城镇职工医保） ③商业医保 ④合作医疗

填表次数：第 次 填表日期： 年 月 日

（以下几项由医生填写）

治疗医院________________________ 治疗科室_________________

临床诊断________________________ 临床分期_________________

治疗方法_________________________ 临床分型_________________

治疗效果：①痊愈 ②有效 ③好转 ④无变化 ⑤恶化 ⑥其它

审核医生（签名）_______________ 审核日期________________

广东医学院 昆明医科大学

高血压患者生命质量测定量表

QLICD-HY（V2.0）

【指导语】：

这份问卷是从整体上了解您**最近一周**内对自己健康状况的感受，从而方便医生有针对性地采取治疗和康复措施。请认真阅读以下每一个问题，并按照您**自己的感觉或判断**圈出最适合您情况的数字。答案无所谓对错，只要是您的真实感受即可，您所提供的资料将绝对保密。谢谢您的支持与配合！

例如：　**您觉得生活有乐趣吗？**

如果您觉得生活比较有乐趣，就在“4”处打一个圈，如下。

一点也没有	有一点	一般	比较	非常
1	2	3	④	5

	过去一周	非常差	比较差	一般	比较好	非常好
GPH1	您胃口好吗?	1	2	3	4	5
GPH2	您睡眠好吗?	1	2	3	4	5
GPH4	您的大便正常吗？	1	2	3	4	5
GSO2	您和家人的关系好吗?	1	2	3	4	5
GSO3	您和朋友的关系好吗？	1	2	3	4	5

	过去一周	一点也没有	有一点	一般	比较	非常
GPH3	您觉得生病或治疗影响您的性功能了吗？	1	2	3	4	5
GPH5	您有疼痛或其他不舒服的感觉吗？	1	2	3	4	5
GPH9	您感到容易疲乏吗？	1	2	3	4	5
GPS2	疾病使您的记忆力下降了吗？	1	2	3	4	5
GPS3	您觉得生活有乐趣吗？	1	2	3	4	5

© 版权所有

	过去一周	一点也没有	有一点	一般	比较	非常
GPS4	您感到烦躁或易怒吗？	1	2	3	4	5
GPS5	您担心被家人视为家庭负担吗？	1	2	3	4	5
GPS6	您担心自己的健康状况变糟吗？	1	2	3	4	5
GPS7	您感到情绪低落或忧伤吗？	1	2	3	4	5
GPS8	您感到悲观失望吗？	1	2	3	4	5
GPS9	您对自己的疾病感到恐惧吗？	1	2	3	4	5
GPS11	疾病使您的脾气（性格）变坏了吗？	1	2	3	4	5
GS06	患病及治疗造成您家庭经济困难了吗？	1	2	3	4	5
GS07	生病及治疗影响您工作或劳动中的地位或作用了吗?	1	2	3	4	5

	过去一周	完全不能	有一点能	一般能	多数能	完全能
GPH6	您能料理自己的日常生活（如：吃饭、穿衣、洗漱、上厕所）吗?	1	2	3	4	5
GPH7	您能劳动（如：做家务、上班或务农等）吗？	1	2	3	4	5
GPH8	您能独立行走吗？	1	2	3	4	5
GPS1	您做事情时能集中注意力吗？	1	2	3	4	5
GPS10	您能够积极乐观地看待自己的疾病吗？	1	2	3	4	5
GS01	您能像生病前一样与别人来往吗?	1	2	3	4	5
GS04	您能得到家庭的关心或支持吗？	1	2	3	4	5
GS05	您能得到家人以外的其他人的关心或支持吗？	1	2	3	4	5
GS08	您能承担相应的家庭角色（如父母、子女、夫妻）吗?	1	2	3	4	5

© 版权所有

过去一周内		一点也没有	有一点	一般	比较	非常
HY1	您有头痛吗？	1	2	3	4	5
HY2	您有头昏眼花吗？	1	2	3	4	5
HY3	您有耳鸣吗？	1	2	3	4	5
HY4	您有心慌吗？	1	2	3	4	5
HY5	您有气短或气促(呼吸困难)吗？	1	2	3	4	5
HY6	您有下肢或脚腕部位水肿吗？	1	2	3	4	5
HY7	您有一侧肢体无力、麻木或活动不灵吗？	1	2	3	4	5
HY8	您有胸口痛或肩背痛吗?	1	2	3	4	5
HY9	您服药后有口干或刺激性干咳吗？	1	2	3	4	5
HY10	您有视力减退或视物模糊吗？	1	2	3	4	5
HY11	您因为需要经常服药或测血压而烦恼吗？	1	2	3	4	5
HY12	您服药后有面部发红和发烫吗?	1	2	3	4	5
HY13	您能适应因病造成的饮食限制或戒烟等生活方式的改变吗？	1	2	3	4	5

© 版权所有
